# Association Between FSIP2 Mutation and an Improved Efficacy of Immune Checkpoint Inhibitors in Patients With Skin Cutaneous Melanoma

**DOI:** 10.3389/fmolb.2021.629330

**Published:** 2021-05-24

**Authors:** Haoxuan Ying, Anqi Lin, Junyi Liang, Jian Zhang, Peng Luo

**Affiliations:** ^1^Department of Oncology, Zhujiang Hospital, Southern Medical University, Guangzhou, China; ^2^Southern Medical University, Guangzhou, China

**Keywords:** skin cutaneous melanoma, Fibrosheath interacting protein 2, immune checkpoint inhibitor, tumor mutation burden, regulatory T cell

## Abstract

**Background:**

Immune checkpoint inhibitors (ICIs) have shown remarkable success in treating skin cutaneous melanoma (SKCM); however, the response to treatment varies greatly between patients. Considering that the efficacy of ICI treatment is influenced by many factors, we selected the Fibrosheath interacting protein 2 (FSIP2) gene and systematically analyzed its potential to predict the efficacy of ICI treatment.

**Methods:**

Patient data were collected from an ICI treatment cohort (*n* = 120) and a The Cancer Genome Atlas (TCGA)-SKCM cohort (*n* = 467). The data were divided into an FSIP2-mutant (MT) group and FSIP2-wild-type (WT) group according to FSIP2 mutation status. In this study, we analyzed the patients’ overall survival rate, tumor mutational burden (TMB), neoantigen load (NAL), copy number variation (CNV), cell infiltration data and immune-related genes. We used gene set enrichment analysis (GSEA) to delineate biological pathways and processes associated with the efficacy of immunotherapy.

**Results:**

The efficacy of ICI treatment of SKCM patients with FSIP2 mutation was significantly better than that of patients without FSIP2 mutation. The patients in the FSIP2-MT group had higher tumor immunogenicity and lower regulatory T cell (Treg) infiltration. Results of GSEA showed that pathways related to tumor progression (MAPK and FGFR), immunomodulation, and IL-2 synthesis inhibition were significantly downregulated in the FSIP2-MT group.

**Conclusion:**

Our research suggests that the FSIP2 gene has the potential to predict the efficacy of ICI treatment. The high tumor immunogenicity and low Treg levels observed may be closely related to the fact that patients with FSIP2-MT can benefit from ICI treatment.

## Introduction

Skin cutaneous melanoma (SKCM) is a common skin tumor caused by uncontrolled proliferation of epidermal melanocytes that is known to have a rapid progression and poor prognosis. According to the 2018 global cancer statistics (Bray et al., 2018), melanoma accounts for approximately 21.6% of new cases of skin cancer and 46% of all skin cancer deaths. Although traditional treatments, including surgery, radiotherapy and chemotherapy, have made great progress in recent years, the efficacy of these traditional treatments is not satisfactory due to the resistance of SKCM to chemotherapy and radiotherapy and the side effects caused by the treatments. The 5-year survival rate is 20% for patients with metastatic melanoma, and the 10-year survival rate is only 10% (Long et al., 2016; Hamid et al., 2019); thus, a more effective treatment is urgently needed.

In recent years, the discovery of CTLA-4, PD-1/PD-L1 and other immune checkpoint molecules has given us a deeper understanding of how immunosuppression limits antitumor immunity and provided new ideas for tumor immunotherapy. Monoclonal antibodies called immune checkpoint inhibitors (ICIs) have been generated to target immune checkpoint molecules. ICIs have been used to treat a variety of malignant tumors, including SKCM, and in 2018, researchers working on them were awarded the Nobel Prize (Peeraphatdit et al., 2020). According to clinical studies, the five-year survival rate of patients with metastatic SKCM treated with an anti-PD-1 monoclonal antibody (nivolumab) is 34%; when an anti-PD-1 antibody and anti-CTLA-4 antibody (nivolumab and ipilimumab) are administered, the five-year survival rate rises to 44% (Larkin et al., 2019). Obviously, patients benefit more from ICIs than from traditional treatment. Although ICIs have shown good clinical efficacy, only a small number of patients benefit from long-term treatment (Rotte et al., 2015), and the factors affecting the efficacy of ICIs remain unclear.

Fibrosheath interaction protein 2 (FSIP2) is an important part of the fiber sheath, which constitutes the cytoskeletal structure of the main part of the sperm flagellum. The sheath is a scaffold of glycolytic enzymes and signaling proteins and plays an important role in vitality regulation (Litchfield et al., 2016; Martinez et al., 2018). Although expression of FSIP2 is testis-specific (Brown et al., 2003), we have found that FSIP2 has a higher mutation frequency not only in male reproductive system tumors, such as testicular germ cell tumors, but also in Paget disease, liver cancer and other cancers (Zhang et al., 2014, 2019; Litchfield et al., 2015). Despite a lack of studies on the relationship between FSIP2 and cancer, FSIP2 has been shown to not only an important part of AKAP4 but to also influence the function of PKA by docking to AKAP4. AKAP4 is highly expressed in a variety of cancers, and the regulatory subunit PKAI of PKA has also been shown to play important roles in promoting the proliferation and transformation of tumors and the generation of immunosuppressive microenvironments in the tumor microenvironment (TME) (Brown et al., 2003; Hussain et al., 2015; Martinez et al., 2018).

The TME is the cellular environment in which tumors exist and includes peripheral blood vessels, extracellular matrix components, and other non-tumor cell nuclear signaling molecules. The growth and metastasis of tumors are inseparably linked to the TME in which the tumors are located (Hui and Chen, 2015). Several studies have noted that the efficacy of ICIs is related to the infiltration of lymphocytes (e.g., CD8 + T cells; CD4 + T cells) and expression of cytokines (e.g., IFN-γ, IL-2, IL-17) in the TME (Abiko et al., 2015; Garris et al., 2018). We speculate that FSIP2 may regulate expression of PKA by affecting that of AKAP4, which in turn influences immune infiltration in the TME. This process also provides suitable immune targets for immunotherapy, suggesting that the efficacy of ICI treatments may be related to FSIP2 mutations.

Currently, no systematic analysis has been performed to address the relationship between FSIP2 and the efficacy of ICIs in the treatment of SKCM. Thus, we sought to collect and analyze existing retrospective ICI-treated cohort data to clarify the association between them. We divided our patients into two groups according to FSIP2 gene mutation and systematically compared tumor immunogenicity, the TME, expression of immune-related genes and signaling pathways between tumors with mutant FSIP2 (FSIP2-MT) or wild-type FSIP2 (FSIP2-WT), providing a theoretical basis for formulating new treatment options.

## Materials and Methods

### Clinical Cohorts and Gene Expression Data

To evaluate the relationship between FSIP2 gene mutation and the efficacy of SKCM patients who received immune therapy, we collected clinical and whole-exon sequencing (WES) data from two clinical cohorts of SKCM patients treated with ICIs from dbGap(phs001041.v1.p1; phs000452.v3.p1). The data from these two datasets were also used in another study (Miao et al., 2018). A total of 120 SKCM patients who received ICI treatment (anti-CTLA-4 therapies; anti-PD-1/PD-L1 therapies; or combined therapies) in the two data sets were included in the ICI treatment cohort for further analysis. We used R package TCGAbiolinks (Colaprico et al., 2016) to download clinical data (including overall survival) and somatic mutation data for a cohort from The Cancer Genome Atlas (TCGA)-SKCM (*n* = 467) from Genomic Data Commons Data Portal^1^, but only some of the patients we collected from TCGA received immunotherapy. Furthermore, we performed immune cell infiltration analysis of gene expression data of the cohort TCGA-SKCM downloaded through TCGAbiolinks. To analyze tumor immunogenicity more completely, we collected neoantigen load (NAL) data from the cohort TCGA-SKCM (Thorsson et al., 2018). Finally, we downloaded the WES data of the SKCM cell line from Genomics of Drug Sensitivity in Cancer (GDSC) for drug sensitivity analysis (Yang et al., 2012).

### Kaplan-Meier Analysis

We included patients from the ICI-treated cohort and the cohort TCGA-SKCM. The patients were grouped into mutant FSIP2 (FSIP2-MT) and wild-type FSIP2 (FSIP2-WT) groups according to FSIP2 gene mutation. Kaplan-Meier (KM) analysis was performed to determine whether there is a difference in the overall survival (OS) of the two groups of patients in the ICI-treated cohort. To determine the impact of FSIP2 mutations on disease development without considering ICI treatment, we also used KM analysis to analyze the OS and disease-free survival (DFS) data of patients in the cohort TCGA-SKCM we collected based on the FSIP2 gene mutation status.

### Tumor Immunogenicity Analysis

The occurrence and development of cancer are always accompanied by changes in DNA. The number of non-synonymous mutations (Mb) in each trillion bases is the tumor mutational burden (TMB), which has been suggested to be related to the clinical efficacy of ICI treatment (Huang et al., 2020). Neoantigens are new peptides produced by somatic mutation. These newly generated peptides can drive immune responses against cancer cells by being recognized as foreign substances in the body (Barroso-Sousa et al., 2020). Therefore, the neoantigen load (NAL) is also considered to be the basic determinant of the immunotherapy response. Data on TCGA-SKCM have been reported in the literature (Thorsson et al., 2018). Consistent with other studies (Chalmers et al., 2017), we used non-synonymous mutations in an ICI-treated cohort (Miao et al), TCGA-SKCM and GDSC-SKCM as raw mutation counts and divided by 38 Mb to quantify the TMB. Based on the R package ComplexHeatmap (Gu et al., 2016), we visualized the mutation panorama and clinical characteristics of the immunotherapy and TCGA-SKCM cohorts (the top 20 mutated genes).

### Copy Number Alteration Analysis

Genomic Identification of Significant Targets in Cancer (GISTIC) is an algorithm used to identify mutation sites that may be associated with cancer pathogenesis. It can be used to visualize regions in the genome to show amplification and missing bases in thousands of samples (Zheng et al., 2020). In this study, we used the Broad GDAC Firehose^2^ to download the Affymetrix SNP 6.0 microarray data (hg19; germline/potential false-positive calls were removed) of TCGA-SKCM. We used GenePattern (Reich et al., 2006) to analyze the downloaded Copy Number Alteration (CNV) segments. The R package Maftools was used to visualize the CNV results obtained from the GISTIC2.0 analysis (Mayakonda et al., 2018). When performing GISTIC2.0 analysis, except for the confidence level set at 0.99 and not excluding the X chromosome before analysis, the GISTIC2.0 analysis used the default (default settings) parameter settings.

### Tumor Immune Status and Drug Sensitivity Analyses

The effectiveness of immunotherapy is affected by multiple factors, such as tumor immunogenicity and antigen presentation efficiency, so we used CIBERSORT (Newman et al., 2015)^3^ to analyze the gene expression matrix of SKCM cohort downloaded from TCGA. We mainly analyzed the infiltration statuses of 22 types of immune cells, including B cells, NK cells, T cell subsets (CD8 + T cells, CD4 + T cells, T helper cells, regulatory T cells (Tregs), and gamma delta T cells), monocytes and macrophages (M0, M1, and M2). In addition, we compared mRNA expression levels of immune-related genes in the FSIP2-MT and FSIP-WT groups created from the cohort TCGA-SKCM. Immune cell-related genes (Hao et al., 2018), immune-related genes and their functional classification (Thorsson et al., 2018) have been reported in the literature. The expression levels of these genes were quantified as log2 (FPKM + 1), and the fold-change (FC) cutoff was selected to be greater than 1.49 or less than 0.67. To analyze the effect of FSIP2 gene mutation on conventional treatment, we downloaded the data for SKCM cell lines with drug sensitivity data from GDSC and compared differences in the sensitivities to different drugs between the FSIP2-MT and FSIP-WT groups.

### Gene Set Enrichment Analysis

Through the R package EdgeR (Robinson et al., 2010), we performed Gene Set Enrichment Analysis (GSEA) on gene expression data (raw count) in the queue for TCGA-SKCM downloaded from TCGAbiolinks. We used the clusterProfiler R package (Yu et al., 2012) to annotate the gene dataset, and *p* < 0.05 was used as the threshold for gene ontology (GO) terms, Kyoto Encyclopedia of Genes and Genomes (KEGG) results and Reactome results to be considered significantly different.

### Statistical Analysis

In this study, R software (version 3.6.1) was used for statistical analysis of data, and all statistical tests were set as two-sided tests. *P* < 0.05 was considered significant. We used the Mann-Whitney *U* test to compare the differences in the TMB, immune cell abundance, immune-related gene expression, and age between the FSIP2-WT and FSIP2-MT groups. Fisher’s exact test was used to compare differences between the top 20 mutation rates of the FSIP2-WT and FSIP2-MT groups in the immunotherapy and TCGA-SKCM cohorts. In the immunotherapy cohort, we used Fisher’s exact test to compare sex and treatment response between the FSIP2-WT and FSIP2-MT groups. In the cohort TCGA-SKCM, we also used Fisher’s exact test to compare sex, race, ethnicity and clinical stage between the FSIP2-WT and FSIP2-MT groups. The KM method and log-rank test were used for survival analysis. The visualization box plot in this paper was generated with the R package ggpurb (Kassambara, 2018), and the CNV visualization false discovery rate (FDR) was 0.05.

## Results

### The Efficacy of ICI Treatment in Patients With FSIP2 Mutation Is Better

**FIGURE 1:**
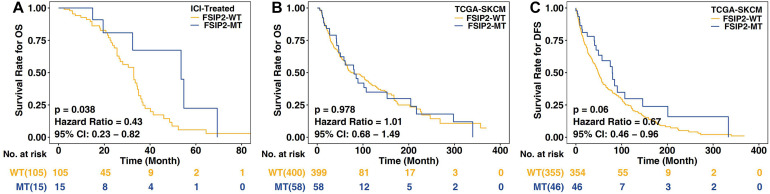
Kaplan-Meier analysis of SKCM patients in the ICI-treated and TCGA-SKCM cohorts. The acquired cohorts were grouped according to the mutation status of FSIP2, with yellow indicating the FSIP2-WT group and blue indicating the FSIP2-MT group. **(A)** Kaplan-Meier curve of the overall survival (OS) of SKCM patients receiving ICIs. The overall survival of the FSIP2-MT group (*n* = 15) was significantly longer than that of the FSIP2-WT group (*n* = 105) [*p* = 0.038; hazard ratio (95% CI): 0.43 (0.23–0.82)]. **(B)** Kaplan-Meier curve of the overall survival of SKCM patients in the collected TCGA-SKCM cohort. The FSIP2-MT group (*n* = 58) and FSIP2-WT group (*n* = 399) had no significant difference in overall survival [*p* = 0.978; hazard ratio (95% CI): 1.01 (0.68–1.49)]. **(C)** Kaplan-Meier curve of the disease-free survival of SKCM patients in the collected TCGA-SKCM cohort. The FSIP2-MT group (*n* = 58) and FSIP2-WT group (*n* = 399) had no significant difference in overall survival [*p* = 0.06; hazard ratio (95% CI): 0.67 (0.46–0.96)].

We divided the somatic mutation and survival data (OS) from the clinical ICI-treated cohort (Miao et al., 2018) (*n* = 120) and TCGA-SKCM cohort (*n* = 467) into two groups based on the FSIP2 mutation status. In this manner, the ICI-treated cohort was divided into FSIP2-MT (*n* = 15) and FSIP2-WT (*n* = 105) groups, and TCGA-SKCM was divided into FSIP2-MT (*n* = 58) and FSIP2-WT (*n* = 409) groups. Survival analysis of these data showed that among the patients receiving ICIs, those with the FSIP2 gene mutation were more sensitive to ICI treatment [*p* = 0.038; hazard ratio (95% confidence interval (CI)): 0.43 (0.23–0.82); Figure 1A]. However, traditional SKCM treatment options are generally surgical resection or chemotherapy (Coit et al., 2019). The survival analysis results for TCGA-SKCM showed that, regardless of whether the patient received ICI treatment, neither OS (*p* = 0.978; hazard ratio (95% CI): 1.01 (0.68–1.49); Figure 1B) nor DFS (*p* = 0.06; hazard ratio (95% CI): 0.67 (0.46–0.96); Figure 1C) was significantly different between the FSIP2-MT and FSIP2-WT groups, demonstrating that the FSIP2 gene mutation itself does not affect the patient’s OS and DFS.

### Relationship Between Clinical Characteristics, Gene Mutations and FSIP2 Gene Mutations in Patients

**FIGURE 2:**
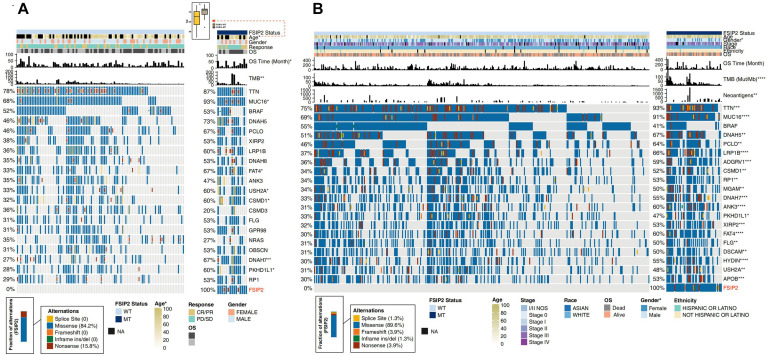
Clinical characteristics and gene mutation panoramas of SKCM patients in the ICI-treated and TCGA-SKCM cohorts. The acquired ICI-treated and TCGA-SKCM cohorts were divided into an FSIP2-MT group and FSIP2-WT group according to the FSIP2 mutation status. **(A)**. We performed Fisher’s exact test on the FSIP2-WT and FSIP2-MT groups of the ICI-treated cohort based on age, sex, treatment response, overall survival and TMB. Except for a higher average age, longer OS time and higher TMB in the FSIP2-MT group, there were no significant differences between the two groups. A comparative analysis of the top 20 mutated genes showed that there were significant differences between the two groups in the mutation frequencies of the MUC16, FATA4, USH2A, CSMD1, DNAH7, and PKHD1L1 genes. The mutation types of the FSIP2 gene were mainly missense mutations (84.2%) and nonsense mutations (15.8%). **(B)**. We performed Fisher’s exact test on the FSIP2-WT and FSIP2-MT groups of the TCGA-SKCM cohort based on age, sex, disease stage, ethnicity, race, overall survival, TMB and neoantigen load. Except for patient sex, TMB and NAL, no parameters showed significant differences between the two groups. A comparative analysis of the top 20 mutated genes showed that, except for the BRAF gene, none of the remaining 19 genes exhibited significant differences in mutation status. The main mutation type of the FSIP2 gene was missense mutation (89.6%), and the other types were splice site (1.3%), frame shift (3.9%), inframe ins/del (1.3%) and nonsense (3.9%) mutations (*p < 0.05; **p < 0.01; ***p < 0.001; ****p < 0.0001).

Based on the mutation status of FSIP2, we compared differences in clinical characteristics between the FSIP2-MT and FSIP2-WT groups. Figure 2A shows that in the ICI-treated cohort, there was no significant difference in sex, treatment response or OS between the FSIP2-WT and FSIP2-MT groups, except for age. Patients with FSIP2 gene mutations tended to have an older age in the ICI-treated cohort (*p* = 0.047). As shown in Figure 2B, in the cohort TCGA-SKCM, there were no significant differences in age, stage, race or ethnicity between the two groups, except for gender. The proportion of men in the FSIP2-MT group was higher than that in the FSIP2-WT group (*p* = 0.043).

Furthermore, we also displayed the gene mutation panoramas of the ICI-treated and TCGA-SKCM cohorts in Figure 2. As shown in Figure 2A, among the top 20 mutated genes in the ICI-treated cohort, except for the higher mutation frequencies of the MUC16, USH2A, DNAH7 and PKHD1L1 genes in the FSIP2-MT group, there were no significant differences in other gene mutations between the two groups. The main FSIP2 mutation types in the ICI-treated cohort were missense (84.2%) and nonsense (15.8%) mutations. Figure 2B shows the 20 genes with the highest mutation rate in TCGA-SKCM. Except for the BRAF gene which was not significantly different between the FSIP2-WT and FSIP2-MT groups, the other genes had a significantly higher mutation frequency in the FSIP2-MT group (*p* < 0.05). The main mutation type in TCGA-SCKM cohort was missense (89.6%); other mutation types, including splice site (1.3%), frameshift (3.9%), inframe ins/del (1.3%) and nonsense (3.9%) mutations, accounted for small percentages of the total mutation rate.

### Patients With FSIP2-MT Have an Elevated TMB and NAL

**FIGURE 3:**
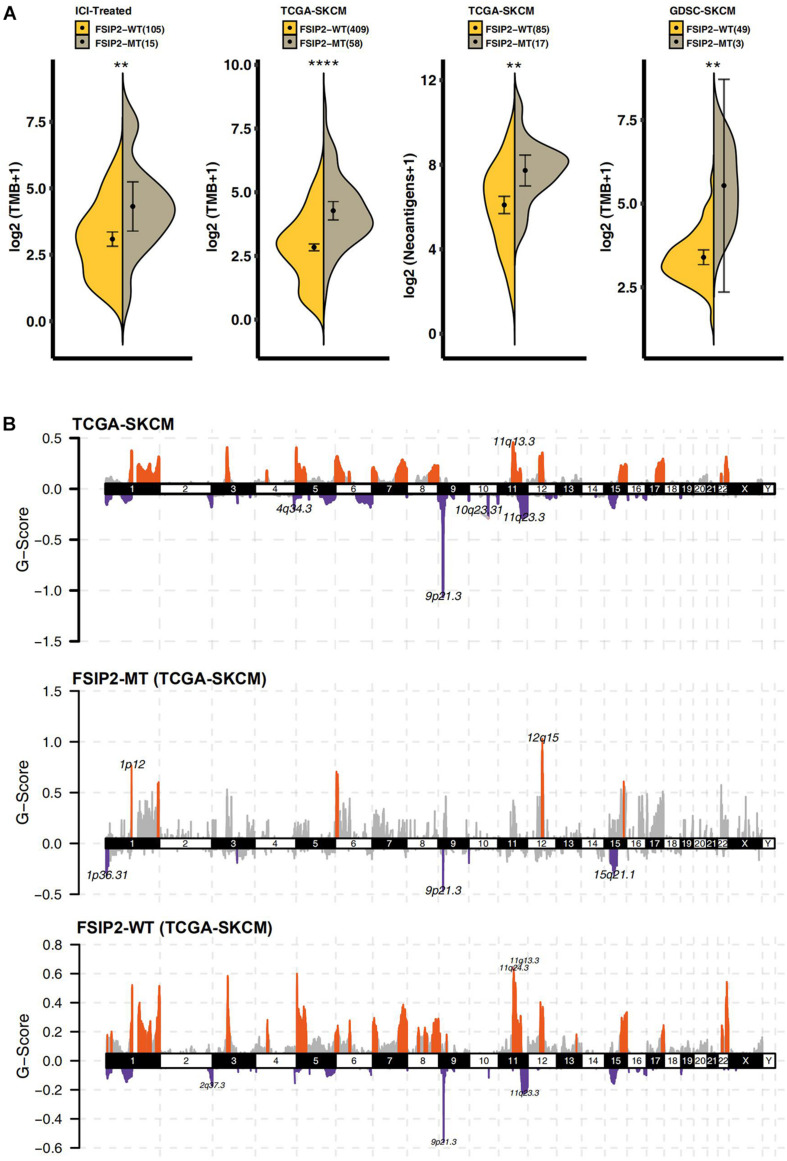
Tumor immunogenicity and CNV analyses of TCGA-SKCM. **(A)** We used the Mann-Whitney *U* test to compare the TMB levels (along the *y*-axis) of the FSIP2-MT group (gray) and the FSIP2-WT group (yellow) in the ICI-treated cohort, TCGA-SKCM cohort and GDSC-SKCM cohort and the tumor neoantigen load (along the y-axis distribution) in the TCGA-SKCM cohort. The numbers in parentheses indicate the total number of patients included in the analysis of each dataset, with *indicating significant differences. The results showed that the FSIP2-MT group had significantly higher TMB levels than the FSIP2-WT group, and the NAL in the FSIP2-MT group was significantly higher. **(B)** The CNV of the TCGA-SKCM cohort was analyzed using GISTIC2.0. We set the *x*-axis as the chromosome number and the *y*-axis as the G-score. The amplified part is displayed above the *x*-axis, and the markedly amplified part is marked with red; the deleted part is displayed below the *x*-axis, and the markedly deleted part is marked with blue (**p < 0.01; ****p < 0.0001).

As shown in Figure 3A, we analyzed the TMB in the ICI-treated cohort and TCGA-SKCM cohort according to the FSIP2 gene mutation status. The results showed that the FSIP2-MT group had a significantly higher TMB than did the FSIP2-WT group. The SKCM cell line data, including WES data downloaded from GDSC (*n* = 52), were also divided into two groups according to the FSIP2 gene mutation status: FSIP2-MT (*n* = 3) and FSIP2-WT (*n* = 49). The TMB levels of the two groups were analyzed, and the results also suggested that the FSIP2-MT group had a higher TMB. The accumulation of mutations in the cancer genome may lead to tumor-specific production of “neoantigens” that are not affected by central T cell tolerance. Therefore, we analyzed the NAL of TCGA-SKCM, and the results showed that the FSIP2-MT group had a higher NAL. The higher TMB and NAL in patients with FSIP2-MT may be related to their better response to ICIs.

### FSIP2-MT Results in a Relatively Low CNV

We analyzed the downloaded TCGA-SKCM queue data by Genomic Identification of Significant Targets in Cancer 2.0 (GISTIC2.0) after grouping according to the mutation status of FSIP2. As shown in Figure 3B, compared with the two copies of chromosomes under normal conditions, TCGA-SKCM samples showed significant amplifications on chromosomes 1, 3 to 8, 11 to 12, 15 to 17 and 22, while deletions were found on chromosomes 1 to 6, 8 to 12, 14 to 16 and 19. In the FSIP2-MT group, the amplified regions were mainly located on chromosomes 1, 6, 12, and 15, and the deleted regions were located on chromosomes 1, 3, 9, and 15. However, the amplification regions in the FSIP2-WT group were mainly located on chromosomes 1, 3 to 9, 11 to 13, 15, to 17, and 22, and the deleted regions were located on chromosomes 1, 3 to 9, 11 to 13, 15 to 17, 19, 22, and X. The distribution and peak value of the amplified/deleted regions in the FSIP2-WT group were significantly higher than those in the FSIP2-MT group, and the results were similar to those of the cohort TCGA-SKCM.

### The Relationship Between FSIP2 and the Tumor Immune Status

**FIGURE 4:**
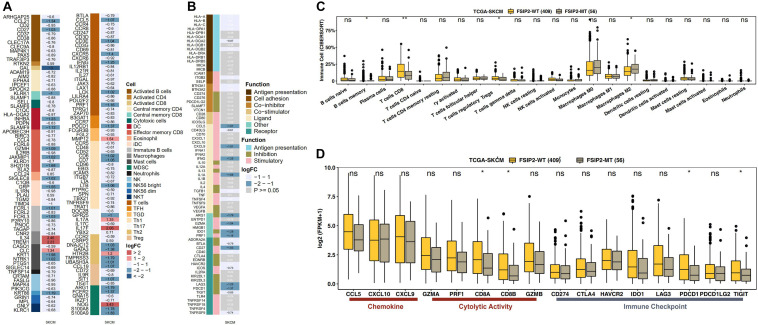
FSIP2 mutation affects tumor immune resistance. **(A)** Heat map of infiltrating immune cells in the FSIP2-MT and FSIP2-WT groups in the SKCM-TCGA cohort. We marked different infiltrating immune cells with different colors. The FC cutoff was set to be greater than 1.49 or less than 0.67, and row annotation represents the immune cells to which genes belong. **(B)** Immune gene heat maps for the FSIP2-MT and FSIP2-WT groups in the SKCM-TCGA cohort. Similarly, the FC cutoff was selected to be greater than 1.49 or less than 0.67, and the row annotation represents the function of the gene. Genes shown in black font have FC > 1.49 or < 0.67 and *p* < 0.05; genes shown in white font were not significantly different between the two groups. **(C)** The TCGA-SKCM cohort was grouped according to the FSIP2 mutation status, with yellow for the FSIP2-WT group and gray for the FSIP2-MT group. The Mann-Whitney U test was used to analyze differences in the infiltration levels of 22 types of immune cells between the two groups, and the results with significant differences are marked with *. **(D)** Immune-related genes were analyzed according to functional classification (chemokine, cytolytic activity, and immune checkpoint) using the Mann-Whitney *U* test. The results with significant differences are marked with * (*p < 0.05; **p < 0.01).

The effect of ICI therapy depends not only on the immunogenicity of the tumor itself but also on the immune status of the tumor. The infiltration of immune cells, such as CD8 + T cells, Tregs, NK cells and macrophages (M0, M1, and M2), also affects the efficacy of ICI treatment. As shown in Figures 4A,B, we analyzed the statuses of infiltrating immune cells and immune genes between the FSIP2-WT group and the FSIP2-MT group in the cohort TCGA-SKCM and marked the cells and genes with significant differences. In addition, we analyzed the infiltration statuses of several specific immune cell populations. As shown in Figure 4C, CIBERSORT analysis results for the FSIP2-MT and FSIP2-WT groups in TCGA-SKCM showed that the memory B cells, CD8 + T cells, and Tregs were significantly upregulated in the FSIP2-WT group, and M2 macrophages were significantly upregulated in the FSIP2-MT group. Furthermore, there was no significant difference in the infiltration of other immune cells between the two groups.

### The Relationship Between FSIP2 and Expression of Immune-Related Genes

As the immune status of tumors is regulated by immune-related genes, expression of these genes affects the efficacy of ICI therapy. According to the immune-related gene sets reported in the literature, we evaluated expression of these genes between the FSIP2-MT and FSIP2-WT groups in TCGA-SKCM. As shown in Figure 4D, expression levels of the CD8A and CD8B genes, which are related to immune cell activity (cytolytic activity), in the FSIP2-WT group were significantly increased, as were expression levels of the PDCD1 and TIGIT genes, which are related to immune checkpoints. In addition, there was no significant difference in the genes responsible for chemokine expression (CCL5, CXCL10, and CXCL9), immune cell activity regulation (GZMA, PRF1, and GZMB) or immune checkpoint regulation (CD274, CTLA-4, HAVCR2, IDO1, LAG3, and PDCD1LG2) between the FSIP2-MT and FSIP2-WT groups.

### The Effect of FSIP2 on Chemotherapy Sensitivity

**FIGURE 5:**
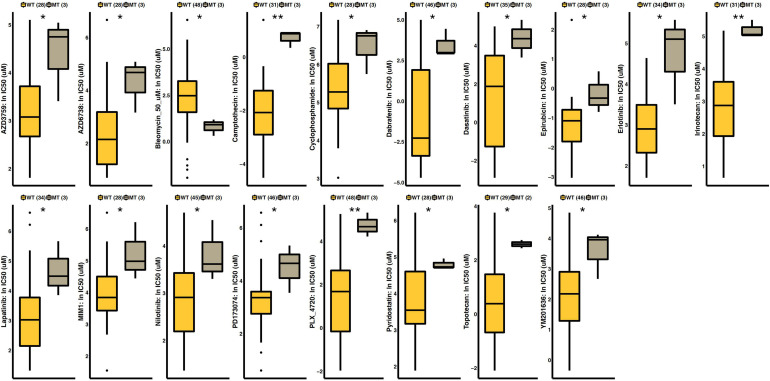
Drug sensitivity analysis of GDSC-SKCM cell line data. SKCM cell lines with drug sensitivity data obtained from GDSC were grouped according to the FSIP2 mutation status, with yellow for the FSIP2-WT group and gray for the FSIP2-MT group. We used the Mann-Whitney U test to analyze the differences in the IC50 values of conventional chemotherapeutic drugs between the FSIP2-MT and FSIP2-WT groups, and the results with significant differences are marked with * (*p < 0.05; **p < 0.01).

As shown in Figure 5, we analyzed SKCM cell line drug sensitivity data obtained from GDSC. After grouping the data according to the mutation status of FSIP2, we compared the difference in the 50% inhibitory concentration (IC50) between the FSIP2-MT and FSIP2-WT groups for 18 commonly used antineoplastic drugs. The results showed that except for that of bleomycin, the IC50s of the FSIP2-MT group were significantly higher than those of the FSIP2-WT group for the other 17 antineoplastic drugs.

### GSEA Analysis Between FSIP2-MT and FSIP2-WT

**FIGURE 6:**
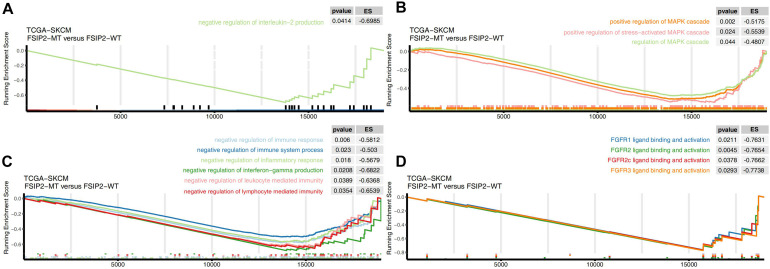
Gene set enrichment analysis delineates biological pathways and processes associated with the efficacy of immunotherapy in TCGA-SKCM. GSEA validated the decreased activity of **(A)** the IL-2 synthesis negative regulation pathway, **(B)** the MAPK activation pathway, **(C)** the negative immune regulation pathway, and **(D)** the FGFR activation pathway in the FSIP2-MT group.

After GSEA of the cohort TCGA-SKCM, we screened out significantly upregulated or downregulated pathways that may be related to the efficacy of ICI treatment. The results are shown in Figure 6. The pathways related to tumor progression, such as positive regulation of the MAPK cascade and FGFR (FGFR1, FGFR2, FGFR2c, FGFR3, and FGFR3c) ligand binding and activation, were significantly downregulated in the FSIP2-MT group (ES < 0, *p* < 0.05), suggesting a better efficacy. We also observed that the negative immune-regulation pathways, such as negative regulation of IL-2 production, negative regulation of the immune response, and negative regulation of lymphocyte-mediated immunity, were significantly downregulated in the FSIP2-MT group.

## Discussion

In this study, we found that FSIP2 gene mutations may affect the efficacy of ICI treatment, but the mechanism is still unknown. To explore the possible mechanisms, we systematically analyzed possible factors, including tumor immunogenicity (the TMB and NAL), CNV, the TME and immune-related gene expression, in the FSIP2-MT and FSIP2-WT groups. The results showed that the FSIP2-MT group had higher immunogenicity (a significantly higher TMB and NAL) and fewer immunosuppressive cells (Tregs). In addition, GSEA of the cohort TCGA-SKCM showed that pathways related to immunosuppression and tumor progression were significantly downregulated in the FSIP2-MT group. These characteristics may all contribute to the result that patients with the FSIP2 gene mutation benefit more from ICI treatment.

BRAF mutations are among the most common gene mutations in SKCM patients; indeed, nearly 50% of SKCM patients carry this mutation. This gene mutation has clinical significance that guides treatment. Studies have shown that BRAF-targeted kinase inhibitors are effective for patients with BRAF gene mutations (Zaman et al., 2019), and the combined use of BRAF and MEK inhibitors has been approved for the treatment of advanced melanoma patients with BRAF V600e mutations (Kakadia et al., 2018). Some research indicates that BRAF inhibitors may enhance the efficacy of checkpoint inhibitors by regulating the tumor immune microenvironment, which may have an impact on our research conclusions (Croce et al., 2019). Therefore, we analyzed the BRAF gene mutation in the ICI treatment and TCGA-SKCM cohorts. We found that there was no significant difference in the BRAF gene mutation in the FSIP2-WT and FSIP2-MT groups. Although we have determined the impact of the BRAF gene mutation itself, we did not assess the use of BRAF-targeting inhibitors in patients, which may have an impact on the results of our final analysis.

Immunogenicity refers to the ability to promote the body’s immune response (Blankenstein et al., 2012). Considering that tumor immunogenicity is affected by a variety of factors, we analyzed the TMB, NAL and CNV data we collected from the ICI-treated and TCGA-SKCM cohorts to systematically evaluate the immunogenicity of SKCM. Previous studies have shown that patients with a high TMB benefit more from ICI treatment (Snyder et al., 2017; Samstein et al., 2019), but there is no clear conclusion regarding whether the TMB can be used as an indicator for screening patients who are sensitive to ICIs (Hellmann et al., 2019). In addition, closely related to the TMB, the NAL generated by cells with somatic mutations can predict the efficacy of ICI treatment (Kim et al., 2020). A study based on 64 SKCM patients also pointed out that there is a strong association between NAL levels and patients’ response to CTLA-4 blockade treatment. They suggested that patients with high NAL can benefit more from immunotherapy (Darvin et al., 2018). Therefore, in this study, we assumed that high NAL is related to better efficacy of immunotherapy. However, CNV has been shown to have potential predictive value for immunotherapy in recent years (Liu et al., 2019). Existing research results indicate that the correlation between CNV and the TMB is weak in predicting the therapeutic effect of ICIs. However, both the TMB and CNV have the ability to predict the efficacy of ICI treatment, suggesting that the TMB and CNV are independent prognostic predictors of ICI treatment (Hieronymus et al., 2018; Liu et al., 2019). In our study, there were also significant differences in the distribution and peak value of amplified/deleted regions between the FSIP2-MT and FSIP2-WT groups, suggesting that the patients in these groups responded differently to ICIs.

The occurrence of an immune reaction is not only closely related to tumor immunogenicity but also requires the participation of the antigen presentation process, which is inextricably linked to the TME (Blankenstein et al., 2012; Wang et al., 2019). CIBERSORT analysis results showed that compared with the FSIP2-MT group, the FSIP2-WT group had a higher level of memory B cells, CD8 + T cells, and Tregs. In the heterogeneous tumor microenvironment, T cells play a large role in immune infiltration, including effector T cells, memory T cells and regulatory T cells, and CD8 + T cells play an important cytotoxicity role in the immunotherapy effect (Li et al., 2019). In addition, studies have shown that B cells are enriched in patients who respond well to ICI treatment (Helmink et al., 2020). The results of Cibersort suggest that patients in the FSIP2-WT group should be able to benefit more from immunotherapy, but this is not in accordance with the observed data. We hypothesize that this may be related to the immunosuppressive TME induced by Treg that affects the normal functions of T and B cells. Tregs are a type of cell that specifically functions in immunosuppression, inhibiting the activation and expansion of lymphocytes that are abnormal or hyperreactive (Sakaguchi et al., 2008). Tregs play very important roles in normal physiological and pathophysiological processes, including antitumor and antimicrobial immunity, transplantation, and allergy (Takahashi and Sakaguchi, 2003). However, they can also suppress the body’s immune response to tumors and contribute to the development of an immunosuppressive TME (Elkord et al., 2010). In many tumors, such as ovarian cancer, pancreatic ductal adenocarcinoma, lung cancer, and SKCM, high expression of Tregs is associated with a poor prognosis. Tregs may exert their immunosuppressive activity through inhibitory cytokines (TGF-β, IL-10, and IL-35), immune checkpoint and inhibitory receptors (e.g., CTLA-4, PD-1, TIGIT) and direct cytotoxicity. We generally believe that ICIs achieve antitumor effects by suppressing immune checkpoints and activating cytotoxic T lymphocytes (CTLs) or effector T cells (Teff), but they may also affect Tregs, which are part of the immune system. Recent studies on Tregs have also pointed out that commonly used ICIs, such as anti-CTLA-4 antibodies, can activate Tregs while activating CTLs, and studies of PD-1 checkpoint inhibitors suggest that nivolumab can abrogate the suppressive function of Tregs, which suggests that Tregs also play a vital role in the immunotherapy response (Chaudhary and Elkord, 2016).

After analyzing immune-related genes in TCGA-SKCM, we found that expression of the immune checkpoint genes PDCD1 and TIGIT, which are related to immunosuppression and T cell depletion in tumors, was increased significantly in the FSIP2-WT group (Johnston et al., 2014; Yeong et al., 2019). We speculate that this upregulated immune checkpoint molecule expression is caused by the elevated level of Tregs in the FSIP2-WT group, which creates an immunosuppressive environment. It is generally believed that patients with CD8 + CTLs that express high levels of immune checkpoint molecules, such as CTLA-4 and PD-1, tend to benefit more from ICI therapy than patients with CD8 + CTLs with low checkpoint molecule expression (Daud et al., 2016). Specifically, highly expressed immune checkpoint-related genes, such as PDCD1 and TIGIT, can theoretically provide targets for ICI treatment, suggesting a better efficacy; however, this is contrary to our results.

According to existing studies, cAMP-dependent RI/PKAI activation induced by adenosine or PEG2 is an important mechanism by which Tregs play a role in tumor immunosuppression (Hussain et al., 2015). After collecting data from the ICI-treated cohort and the cohort TCGA-SKCM for analysis, we found that the FSIP2 mutations found in both cohorts were mainly missense mutations. This may cause FSIP2 to lose its original function and further affect Treg-induced PKAI-mediated immunosuppression of antitumor immunity by reducing AKAP4 expression and the attachment of PKA to AKAP4 (Brown et al., 2003; Martinez et al., 2018).

Our GSEA of TCGA-SKCM data also indicated that pathways related to tumor progression (MAPK and FGFR), immunomodulation, and IL-2 synthesis inhibition were significantly downregulated in the FSIP2-MT group. According to existing research results, inhibition of the MAPK pathway achieves good results in tumor immunotherapy, which may be related to downregulation of immunosuppressive factor expression (Deken et al., 2016; Loi et al., 2016). Another study noted that FGFR blockers not only showed good antitumor effects but also improved the effect of immunotherapy when combined with anti-PD-1 therapy (Palakurthi et al., 2019). In clinical studies, the combination of IL-2 and anti-CTLA-4 therapy enhanced the antitumor effect (West et al., 2013; Kohlhapp et al., 2015). The results of GSEA also suggest that the FSIP2-MT group can achieve better ICI treatment efficacy.

## Conclusion

In this study, we found that the efficacy of immunotherapy in the FSIP2-MT group was better than that in the FSIP2-WT group. The FSIP2-MT group had higher tumor immunogenicity and lower Treg levels; GSEA also suggested that the FSIP2-MT group responded better to ICI treatment. We attempted to elucidate the possible mechanism by which FSIP2 mutation and the tumor immune microenvironment affect the efficacy of ICI treatment in SKCM patients. Overall, this work provides theoretical guidance for further improving the efficacy of ICIs in SKCM patients with or without FSIP2 mutations. However, as there are few studies or data on FSIP2 mutations and tumor immunotherapy, the association between FSIP2 mutations and SKCM needs to be verified by further experiments.

## Data Availability Statement

The datasets presented in this study can be found in online repositories. The names of the repository/repositories and accession number(s) can be found in the article/Supplementary Material.

## Author Contributions

JZ and PL conceived and designed the study. HY, AL, and JL performed the experiments. AL contributed significantly to the data analyses. JL was responsible for the revision of the article. HY wrote the manuscript. HY, AL, JL, and PL reviewed and edited the manuscript. All authors read and approved the manuscript.

## Conflict of Interest

The authors declare that the research was conducted in the absence of any commercial or financial relationships that could be construed as a potential conflict of interest.

## Footnotes

1 https://portal.gdc.cancer.gov/

2 http://gdac.broadinstitute.org/

3 http://cibersort.stanford.edu/
